# Supplementary material for: Differential Induction Pattern Towards Classically Activated Macrophages in Response to an Immunomodulatory Extract from Pleurotus ostreatus Mycelium
Source: J Fungi (Basel). 2021 Mar 11;7(3):206. doi: 10.3390/jof7030206 (PMC8000819; doi:10.3390/jof7030206)
Supplement: Supplementary file 1 [file jof-07-00206-s001.zip › Supplementary Material/S1 Llauradó et al 2021 Cytotoxicity Protocol.docx]

**Cytotoxicity in Macrophages (resazurin dye reduction test)**

**Protocol**

**Cell lines**

- RAW 264.7 (mouse leukemia monocyte macrophage/adherent cell line)

1. Cells will be cultivated in flask at 37°C, 5% CO_2_.
2. Remove the cells with cell scrape (only for adherent cells) and centrifuge (130 xg, 10 min, 4°C). Count the cells and adjust the concentration of the cell suspension. The cells (5 x 10^5^cells) in 200 µl will be added into each well of the 96-well plates.
3. Incubate the plates for 24 h at 37°C in 5% CO_2_.
4. Centrifuge the plates (for THP-1, non-adherent cells only) 1800 rpm/10 min/RT
5. Gently discard the supernatant
6. Add 200 µL of fresh medium into the 96 well plates (the extract concentrations and the solvent should be diluted in medium prior). Control cells will be incubated with 200 µL of medium only.
7. Add 10 µL of reference drugs (Ivermectin or Tamoxifen)******
8. Incubate the plates for 24-48 at 37°C in 5% CO_2_.
9. Add 50 µL of resazurin (2,2 µg/mL) to each well and incubated for 4 h at 37°C.
10. Fluorescence will be measured (550 nm excitation and 590nm emission). ***

*Range for plant/mushrooms extracts (1024/512µg/mL – 2 µg/mL). Preparation of extract concentration in the same medium and prior to the experiment is strongly recommended (fold serial dilution).

**See the code, in 4°C fridge (Tamoxifen initial concentration of 64ug/ml)

** Fluorescence will be measured (550 nm excitation and 590nm emission) every 15’ during 4 hours in the first place, until the protocol be set up.

**Interpretation of the Results based on LMPH protocol for cytotoxicity in MRC5-SV2 cells (human lung fibroblasts)**

**IC_50_ values**

| Test model | Activity score based on **IC_50_ values** | | |
| --- | --- | --- | --- |
|  | **1** (inactive) | **2** (moderately active) | **3** (highly active) |
| Cytotoxicity | >30 | <30-10> | <10 |

**Remarks**

- Activity is expressed as IC_50_ values in µg/mL (for extract without exact molecular weight).
- Semi-quantitative activity scores (range 1-3) are given, based on the level of IC_50_.
- Extracts with score-3 require confirmation testing and further follow-up.
