# Supplementary material for: Differential Induction Pattern Towards Classically Activated Macrophages in Response to an Immunomodulatory Extract from Pleurotus ostreatus Mycelium
Source: J Fungi (Basel). 2021 Mar 11;7(3):206. doi: 10.3390/jof7030206 (PMC8000819; doi:10.3390/jof7030206)
Supplement: Supplementary file 1 [file jof-07-00206-s001.zip › Supplementary Material/S4 Llauradó et al 2021 Macrophages polarization.pdf]

TOPIC: Macrophage polarisation and evaluation

AIM:

- *In vitro differentiation of primary bone marrow cells into different macrophage subsets.*
- *Discrimination between classically activated M1 and alternatively activated M2 macrophages, using flow cytometry in conjunction with qRT-PCR.*

Keywords

Macrophage, polarisation, isolation, flow cytometric evaluation, qRT-PCR

1. Collection and preparation primary bone marrow cells

Materials

- ✓ Dissection material (e.g. scalpel, scissors, foreceps)
- ✓ 15 ml Falcon (BD)
- ✓ Filter tips
- ✓ Syringes
- ✓ Needles (25G, 21G)
- ✓ ACK (Ammonium Chloride Potassium solution) (**SOP n° 61**)
- ✓ KOVA counting chamber
- ✓ 6-well, 24-well plate
- ✓ L929 supernatant (SOP n°)

Reference number	Firm	Product	LMPH number
		RPMI 1640	534
		Non-Essential Amino Acids	
		PeniStrep	
P2256	Sigma-Aldrich	Sodium pyruvate	55
		L-glutamine	
		Inactivated Fetal bovine serum	
14190-094	Life Technologies	Phosphate Buffered Saline	536

Methods

- Euthanize mice (CO_2) and disinfect the skin with ethanol. From now on, work under the laminar flow.
- Remove the hind legs.
- Remove most of the skin and muscles until the femur and tibia are completely clean
- Carefully cut off both ends of the bone until a small red dot is seen (= bone marrow) and flush with 1 ml RPMI (25G needle). Repeat until the cavity is white.
- Collect the bone marrow in a sterile 15ml falcon tube and keep on ice.
- To make a single cell suspension, aspirate the cells using a 21G needle and pore the cells through a 25G needle. If necessary, repeat the procedure. Do not repeat this more than 2 times, if this can damage the cells.
- Centrifuge the cells for 10’ @ 2000 rpm, remove the supernatant.
- To lyse the red blood cells, pipet 3 ml ACK on the pellet and resuspend. Stop cell lysis after 3’ by adding excess RPMI to the cells.
- Centrifuge for 10’ @ 2000 rpm, remove the supernatant.
- Rinse the cells 2x with RPMI and dissolve the pellet in RPMI to count the cells with a KOVA® chamber.
- Adjust the cells to the right concentration (10^6 cells/ml), bring in a 6-well (3-5ml/well) or 24-well plate (1ml/well) and let the cells attach for 24h (RPMI without iFBS).
- Change the medium with enriched RPMI medium (+1% NEAA + 1% penistrep + 1% sodium pyruvate +1% L-glutamine + 10% iFBS + 15% L929 supernatant (containing M-CSF)) and incubate the cells for 3 days. The cells have now the M0 phenotype.

2. Polarization macrophages

Materials

- ✓ Eppendorfs
- ✓ Filter tips

Reference number	Firm	Product	LMPH number
L2755	Sigma-Aldrich	Lipopolysaccharide	260
I4777	Sigma-Aldrich	Interferon-gamma	306
404-ML-010	R&D Systems	Interleukin-4	795
413-ML-005	R&D Systems	Interleukin-13	796

Methods

- To differentiate the cells into the M1 phenotype, stimulate the cells with 100 ng/ml LPS (LPS stock: 1 mg → aliquots in -20°C) (+ 25 ng/ml IFN-γ, not necessary) and incubate for 24 or 48 hours. To differentiate the cells into the M2 phenotype, incubate the cells with 25 ng/ml IL-4 (+ 25 ng/ml IL-13, not necessary) and incubate cells for 24 or 48 hours.

3. Flow cytometric evaluation (on ice!)

Materials

- ✓ Eppendorfs
- ✓ Filter tips
- ✓ Flow cytometer

Reference number	Firm	Product	LMPH number
14190-094<br>EDS<br>-	Life Technologies<br>Sigma-Aldrich<br>Bioceros<br>BD	Phosphate Buffered Saline<br>Ethylenediaminetetraacetic acid, EDTA<br>Fc-block (1/200)<br>Antibodies (see antibody list)<br>- CD80-FITC (1/100)<br>- CD86-PECy7 (1/100)<br>- CD150-APC (1/100)<br>- CD200R-PE (1/100)	536<br>136<br>794

Methods (on ice!)

- Remove the supernatant (and store @ -80°C for cytokine measurements). Collect the macrophages by bringing cold 5 mM EDTA (in PBS) to the cells and pipet up and down (blue tip + yellow tip) to detach the cells from their culture plate. Centrifuge 10’ @ 2000 rpm (4°C)
- Wash the cell pellet 2x with PBS. Centrifuge 10’ @ 2000 rpm and discard supernatant. Divide the cells into different vials (according to the number of antibody stainings)
- Bring 50 µl Fc-block (1/200) on the cell pellet and incubate 15’
- Centrifuge 10’ @ 2000 rpm and discard supernatant (no wash)
- Bring 50 µl antibody mix on the cell pellet and incubate for 30’ (in dark!)
- Wash cells 1x with PBS. Centrifuge 10’ @ 2000 rpm
- Resolve the cell pellet in 250 µl PBS and measure the cells on with flow cytometry (room 7.33) (Macrophage templates: desktop Mac) (Settings: FSC: E00, SSC: 390, FL1: 372, FL2: 406, FL3: 398, FL4: 626) (Settings FSC and SSC: depending on cell type)

4. RNA extraction

Materials

- ✓ Eppendorfs
- ✓ Filter tips
- ✓ NanoDrop

Reference number	Firm	Product	LMPH number
14190-094	Life Technologies	Phosphate Buffered Saline	536
EDS	Sigma-Aldrich	Ethylenediaminetetraacetic acid, EDTA	136
74136	Qiagen	RNeasy Plus Mini kit	-

Methods

- Collect the macrophages by bringing cold 5 mM EDTA (in PBS) to the cells and pipet up and down (blue tip + yellow tip) to detach the cells from their culture plate. Centrifuge 10’ @ 2000 rpm (4°C).
- Extract RNA by using the RNease Plus Mini kit (Qiagen) according to the manufacturer’s instructions. Work under the laminar flow (room 7.32).
- Keep samples on ice.
- Determine the concentration with the NanoDrop.
- Bring all samples to same concentration (with PCR water) (not < 70 ng/µl).
- Store samples at -20°C.

5. Generation of cDNA

Materials

- ✓ Eppendorfs
- ✓ Filter tips
- ✓ Heat block (room 7.32)

Reference number	Firm	Product	LMPH number
600180<br>4368814	Aglient Technologies<br>ThermoFisher	AccuScript High Fidelity RT-PCR System<br>High-Capacity cDNA Reverse Transcription kit	718<br>797

Methods

- Thaw all reagents on ice. Make cDNA with the High-Capacity cDNA Reverse Transcription kit or the AccuScript High Fidelity RT-PCR System. If possible, perform all manipulations under the laminar flow (room 7.32)
  High-Capacity cDNA Reverse Transcription kit:
  - Calculate the volume of the components needed
    For 10 µl RNA, 10 µl master mix is needed, always take some extra reactions to make sure you have enough.

Component	Volume (µl) / reaction kit
10x RT buffer	2
25x dNTP Mix (100 mM)	0,8
10x RT random primers	2
MultiScribe Reverse Transcriptase	1
Nuclease-free water	4,2
TOTAL (2x master mix)	10

  - Place the 2x RT master mix on ice and mix gently
  - Pipette 10 µl of 2x RT master mix into each epp
  - Pipette 10 µl of RNA sample into each epp, pipetting up and down 2 times to mix
  - Short spin the samples
  - Incubate 10 min at 25 °C (room temperature)
  - Incubate 120 min at 37 °C (heat block)
  - Incubate 5 min at 85 °C (start counting once it is at 85 °C)
  - Store samples at -20°C.

6. qRT-PCR

Materials

- ✓ Eppendorfs
- ✓ Filter tips (20 µl, 10 µl)
- ✓ MicroAmp® Optical 96-wellreaction plate (Applied Biosystems)
- ✓ MicroAmp® Optical Adhesive Film (Applied Biosystems)

**Reference number**	**Firm**	**Product**	**LMPH number**
4440040	Applied Biosystems	Taqman Universal Master mix, no UNG II	793
4331182 (250rxn)	Applied Biosystems	Taqman primer/probe	
		- hprt	789
		- nos2	790
		- arginase-1	791
		- fizz-1	792
95284-100ML	Sigma	Water for molecular biology (PCR water)	562

Methods

- Thaw all reagents on ice. Make a mix of Taqman Universal Master mix, Taqman primer/probe and PCR water according to the table below. Pipet **15 µl** of this **mix/well** of an optical 96-well reaction plate (MicroAmp®, Applied Biosystems). Use filter tips (new tip/well) and work precise. Include a standard curve for each gene (minimal 5 concentrations). Perform all manipulation in room 7.36, pre-PCR.
- Thaw samples on ice. Add **5 µl cDNA/well** (laminar flow or bench) (no samples in pre-PCR). If possible, all samples need to be done in triplicate (only when you have not enough sample, duplicate is also fine).
- Close the 96-well plate with a seal and centrifuge for 1’ to remove air bubbles at the bottom of the plate (small centrifuge next to PCR device).

	Final concentration	1 reaction
		µl
Taqman Universal Master mix II, no UNG (2x)	1 x	10
PCR water		4
Taqman primer/probe (20x)	1x	1
cDNA	x ng	5
**Total volume**		20

- Open StepOne software and use the qRT-PCR conditions below:
  - denaturation: 10’ @ 95°C
  - annealing and extension: 45x (30’’ @ 95°C and 1’ @ 60°C)
