# Supplementary material for: Differential Induction Pattern Towards Classically Activated Macrophages in Response to an Immunomodulatory Extract from Pleurotus ostreatus Mycelium
Source: J Fungi (Basel). 2021 Mar 11;7(3):206. doi: 10.3390/jof7030206 (PMC8000819; doi:10.3390/jof7030206)
Supplement: Supplementary file 1 [file jof-07-00206-s001.zip › Supplementary Material/S5 Llauradó et al 2021 Phagocytosis assay protocol.docx]

SOP 173: Vitro – Cells – Phagocytosis Author: Marjorie

Version : 1 Date : 8/6/2016

## TOPIC*:* phagocytosis assay

**Keywords**

Phagocytosis, cells, beads

**Materials**

| **Reference number** | **Firm** | **product** |  | **LMPH number** |
| --- | --- | --- | --- | --- |
|  |  | Adherent cell culture |  |  |
| 631-1577 | VWR | Sterile coverslips |  |  |
| 662160 | Greiner | 24-well plate |  |  |
|  |  | Medium with iFCS |  |  |
|  |  | PBS with Ca/Mg |  | **SOP 43** |
|  |  | PBS without Ca/Mg |  | **SOP 43** |
|  |  | 4% paraformaldehyde | 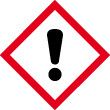 | **SOP 70** |
| L4655-1ML | Sigma-Aldrich | Carboxylate-modified fluorescent beads |  | K721 |
| L1030-1ML | Sigma-Aldrich | Amine-modified fluorescent beads |  | K838 |
| T 9284 | Sigma-Aldrich | Triton-X (end concentration: 0.5 %, diluted in PBS) |  | K50 |
| T7471 | Life Technologies | Texas Red X phalloidin |  | K631, divided in -80 °C.2, box 2.2.20 |
| 32670 | Sigma-Aldrich | DAPI | 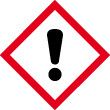 | K107 |
| D27802 | Sigma-Aldrich | 1% DABCO dissolved in 90% glycerol, 10% PBS without Ca/Mg | 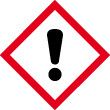 | K579 |
|  |  | Substrate glass |  |  |
|  |  | Transparent nail polish |  |  |

**Methods**

- Place the test tube with coverslips (CS) in the “dry incubator” for 1 h at 180 °C.
- Cool the CS down and they are ready to use.

**Day 0 (sterile):**

1. Place CS in a 24 well plate with a sterile tweezers (sterilizes the tweezers with ethanol, wait until it is dry before you use it).
2. Detach the cells from the culture flask and add them to the CS, final volume for 1 well in a 24-well plate is 1 ml. (Optimize the amount of cells you want on your CS, depending of you experiment. As a guideline, the cells should not be more than 90% confluent at the day of staining.) Or add primary adherent cells to the well plate. Make sure no air bubbles are under the CS.
3. Place the cells in the incubator at 37 °C and 5% CO_2_ for at least 24 h.

**Day of staining (if the cells don’t contain pathogens, this experiment can be done on the bench, otherwise perform the staining until the fixation of the cells in the laminar air flow):**

Always aspirate the liquid in the well plate with a 1 ml pipet or the vacuum pump before adding new solutions

1. Watch the cells under the microscope to make sure the cells look good.
2. Rinse the cells twice with 900 µl medium.
3. Put out the lights before you proceed the experiment because you are using fluorescent beads.
4. Prepare the diluted beads in medium to the cells. Dilute the beads 1 over 500 in medium. For each well, prepare 300 µl bead suspension solution.
5. Add 300 µl beads to the cells.
6. Incubate the beads during 60 min at 37 °C, as a control keep the cells at 4 °C (no phagocytosis).
7. Rinse the cells twice with 900 µl PBS with Ca/Mg.
8. Add 400 µl filtered 4% paraformaldehyde and incubate for 20 min at room temperature (make sure it don’t last more than 20 min).
9. Rinse the cells 3 times with 900 µl PBS without Ca/Mg.
10. Permeabilize cells with 0.5% Triton X-100 (10 minutes, RT) .
11. Wash 3x with 900 µl PBS without Ca/Mg.
12. To visualize the membrane (optional), add Texas-Red phalloidin to the solution. Use a final concentration of 1 U/ml in an end volume of 300 µl diluted in PBS without Ca/Mg for each well.
13. Add 300 µl phalloidin solution to the cells.
14. Incubate for 1 h at 37 °C.
15. Rinse the cells twice with 900 µl PBS without Ca/Mg.
16. Dilute the DAPI to 2.5 µg/ml in a dark epp. For each well, prepare 300 µl DAPI solution. Using DAPI is optional to visualize the nucleus of the cells.
17. Add 300 µl DAPI solution to the cells.
18. Incubate the DAPI for 2 min at room temperature.
19. Rinse the cells 2 times with 900 µl PBS without Ca/Mg.
20. Place a drop of 1% DABCO solution on a substrate glass
21. Use tweezers and a lightly bended needle to remove the CS of the 24-well plate. Try to remove some PBS left on the CS by touching a green cloth gently with the side of the CS.
22. Place the CS on the substrate glass with the cells touching the DABCO solution.
23. Fixate the CS with transparent nail polish at 4 sides, once they are a bit dry, cover the entire perimeter of the CS with nail polish.
24. Keep the substrate glass in an appropriate box at 4 °C until you analyze the cells with fluorescence microscopy.

Instead of fluorescent beads, life or dead pathogens can be used. Added to the cells at the same concentration as the beads. The bacteria can be stained with DAPI.

- *Escherichia coli* (Gram-negative)
- *Streptococcus pneumoniae* (Gram-positive)
- *Mycobacterium tuberculosis* (acid-fast stain)
- *Leishmania infantum*
- *Leishmania donovani*
- *…*
