# Supplementary material for: Differential Induction Pattern Towards Classically Activated Macrophages in Response to an Immunomodulatory Extract from Pleurotus ostreatus Mycelium
Source: J Fungi (Basel). 2021 Mar 11;7(3):206. doi: 10.3390/jof7030206 (PMC8000819; doi:10.3390/jof7030206)
Supplement: Supplementary file 1 [file jof-07-00206-s001.zip › Supplementary Material/S2 Llauradó et al 2021 Griess assay Protocol.doc]

**Nitric oxide (NO) determination by Griess reagent**

**Materials and reagents**

1. RAW 264.7 cell line
2. DMEM + 10% iFBS + glutamine
3. DPBS
4. LPS stock concentration 100 µg/mL (or 1 µg/mL)
5. Griess reagent kit (Invitrogen, Molecular Probe®)
6. L-NAME hydrochloride (Nr.: 312; -20°C),
7. Two 96-well flat-bottom enzymatic assay plate.
8. Samples of plants/mushrooms fractions (stock concentration)

**Method and assay**

- *Stimulation of cells (macrophages) in 96-well plate*

1. Keep the DMEM to 37°C in thermostatic bath.
2. Taw stock LPS
3. Shake LPS +/- 30 min
4. Cells in flask at 37°C, 5%CO_2_. Remove the cells with cells scrape, centrifuge 130 xg-10 min, discard the supernatant and add new medium (fresh).
5. After cell counting, add 200 μL of cells suspension per well in the 1^st^ 96-well plate (cell density: 5 x 10^5^cells/mL).
6. Incubate the plate 24h at 37°C and 5% CO_2_.
7. After 24 h, gently discard the old medium
8. Wash twice with 200 μL of DPBS pre-warmed (gently), using repeat-one dispenser
9. Discard the DPBS and add fresh medium (200 μL)
10. Add LPS (positive control, LPS= 100 ng/mL) (the total volume is 200 µL per well)
11. Add the plant/mushrooms fractions per well (500 and 100 µg/mL of final concentration) (the total volume is 200 µL per well) and the L-NAME (100 – 250 µM)
12. Incubate 24h for stimulation at 37°C and 5% CO_2_.

- *Griess reaction*

1. Firstly, add 130 µL of pure water in each well (2^nd^ new 96-well plate).
2. After 24 h on incubation, take 150 µL of supernatant (from the 1^st^ 96-well plate) and add in each well of the 2nd new 96-well plate. Mix gently and slow.
3. Add 20 µL Griess reagent (Invitrogen kit) (previously prepared in equal amounts)*****. Mix slow
4. Incubate for 30 min (approx.) at room temperature and protected from the light.
5. Measure at 548 nm(550nm) at microplate reader (between 520-590).

***The griess reagent kit should be protected from the light. Prepare the Griess reagent few minutes before step number 3. The kit is provided by Invitrogen.**

**Colored wells represent different plant extracts**

**Remarks**

- The solvents should not exceed 0.1 % of the total volume in wells. Keep in mind prepare the extract concentrations at 300 mg/mL.
- The extract concentrations should be prepared in others vials (or plates) and always diluted with the same medium that will be used. Prepare the concentration some hours prior the experiment.
- Both LPS and L-NAME should be at 100 ng/ml and 100 µM in the wells, respectively.
