# Supplementary material for: Differential Induction Pattern Towards Classically Activated Macrophages in Response to an Immunomodulatory Extract from Pleurotus ostreatus Mycelium
Source: J Fungi (Basel). 2021 Mar 11;7(3):206. doi: 10.3390/jof7030206 (PMC8000819; doi:10.3390/jof7030206)
Supplement: Supplementary file 1 [file jof-07-00206-s001.zip › Supplementary Material/S3 Llauradó et al 2021 EPR Protocol.doc]

# **Protocol Intracellular NO detection in macrophages by Electron Paramagnetic Resonance Spectroscopy (EPR).**

**Reagents:**

RAW 264.7 macrophages in T25 80% confluent

DMEM medium

FeSO_4_.7H_2_O (Sigma – n°138 Balance room/RT)

DETC (Sigma – n°407 Frigde 4°C)

LPS (Frigde 4°C )

IFN-γ (Frigde 4°C)

Mushroom extracts (previously filtered with bacteriological filter 0.2µm)

(HW-P)

**Solutions:**

LPS (stock solution 1 µg/mL)

IFN-γ (stock solution 10 µg/mL)

**Protocol:**

1. **Stimulation of RAW cells:**
1. Stimulate with LPS (100ng/mL), IFN-γ (5 ng/mL) and HW-P (500, 100 µg/mL) for 24 hours in DMEM medium.*****
2. Use fresh medium – then add the stimulators and mushroom extract–to the culture flask in which the macrophages should already have been cultured.*****
3. Incubate this for 24 hours on a 37°C, 5% CO_2_ incubator.

**2.** **Preparation of colloidal Fe(DETC)_2_:**

1. Take 2 x 15 ml Falcon tubes on ice
2. Add 10 ml of KHB (Krebs HEPES Buffer) to each tube.
3. Bubble with N_2_ gas for 30 minutes.
4. Add 4,5 mg of FeSO_4_.7H_2_O to one Falcon
5. Add 7,2 mg DETC to the other one
6. Bubble for 20 minutes with N_2_ gas
7. When all of the Fe is dissolved, add as soon as possible 1 ml of this solution (500µl from each tube) to the cells (put directly into the medium) (the reaction will start immediately: copper particles will form).
8. Incubate for 1 hour
9. Remove the medium up to 1 ml
10. Scrape the cells

**3.	ESR measurement**

1. Remove the end of a 1 ml syringe
2. Add 500 µL of the cell suspension to the syringe
3. Freeze it immediately in N_2_ – keep it frozen in -80°C or on N_2_

**ESR Settings:**

B0: 3290 G

Sweep: 80G

Mod amplitude: 5000 mG

Gain: 5E2

MW atten: 10

***Design (5 culture flask, T25):**

**F1:** Only cells

**F2:** Cells + LPS + INF-γ

**F3:** Cells + HW-P (500 µg/mL)

**F4:** Cells + HW-P (100 µg/mL)

**F5:** Only DMEM medium

**F6:** DMEM + H_2_O
