# Supplementary material for: Differential Induction Pattern Towards Classically Activated Macrophages in Response to an Immunomodulatory Extract from Pleurotus ostreatus Mycelium
Source: J Fungi (Basel). 2021 Mar 11;7(3):206. doi: 10.3390/jof7030206 (PMC8000819; doi:10.3390/jof7030206)
Supplement: Supplementary file 1 [file jof-07-00206-s001.zip › Supplementary Material/S6 Llauradó et al NF-KB reporter assay Protocol.doc]

**Protocol to determine the NF-κB reporter gene *in vitro* using**

**U937 monocyte cell line (3xB-LUC cells)**

**Materials**

1. U937 monocyte cell line (3xB-LUC cells)
2. RPMI + 10% iFBS + glutamine
3. DPBS
4. LPS stock concentration 100 µg/mL (or 1 µg/mL)
5. PMA 10 ng/mL (Phorbo 12-Myristate 13-Acetate)
6. Hygromycin (75 µg/mL)
7. 24-well and 96-well flat-bottom enzymatic assay plates.
8. Samples mushroom fractions (stock solution 1 or 2 mg/mL)
9. Steady-Glo® Luciferase Assay System kit (<https://be.promega.com/products/reporter-assays-and-transfection/reporter-assays/steady-glo-luciferase-assay-system/?catNum=E2510>)

**Method and assay**

- *Sub-culturing of U937 cells*

1. Keep the RPMI to 37°C in thermostatic bath.
2. Taw stock U937 vial by gentle agitation in a 37°C water bath or room temperature (Thawing should be rapid.
3. Transfer the vial content to a centrifuge tube containing 9.0 mL complete culture medium (RPMI) and spin at 130 x g for 10 minutes, 4°C.
4. Resuspend cell pellet with the recommended complete medium (RPMI) and dispense into a 25 cm^2^ or T25 culture flask), add Hygromycin antibiotic (75 μg/mL).
5. Incubated the cells in flask at 37°C, 5%CO_2_.
6. Fot subculturing and Medium Renewal, add fresh medium every 2-3  days (depending on cell density)

- *Stimulation of U937 cells*

1. Transfer the cell culture to conical 50 mL tube and centrifuge to 130 x g for 10 minutes, 4°C.
2. Determine the cell density and adjust to 5x10^5^cell/mL, and suspend the cells in new RPMI medium without antibiotic.
3. Add 200 µL of cells suspension per well in the 96-well plate (5 x 10^5^cells/mL) and add PMA (10 ng/mL), incubate 24h at 37°C and 5% CO_2_. After this period replace the RPMI for new medium without PMA (wash twice with 200 μL of DPBS, gently, see if it is necessary)
4. Add LPS (LPS= 100 ng/mL as positive control) and mushrooms sample (100, 250 and 500 µg/mL, final volume of 200 µL per well)
5. Incubate 24h for stimulation at 37°C and 5% CO_2_.

- *Steady-Glo® Luciferase Assay System*

1. Remove 96- well plates containing mammalian cells from the incubator. The **plates used must be compatible with the luminometer** being used. For best results, equilibrate cultured cells to room temperature before performing Step 2.
2. To each plate well, add a volume of Steady-Glo® Reagent equal to the volume of culture medium in the well, and mix. (For 96-well plates, typically 100µl of reagent is added to cells grown in 100µL of medium) (Note: Discard 100 µL of supernatant prior to start this step).
3- Wait at least 5 minutes to allow cell lysis, then measure luminescence in a Luminometer microplate reader.

**Reagent Preparation**

1. To prepare the Steady-Glo® Reagent, transfer the contents of one bottle of Steady-Glo® Buffer to one bottle of Steady-Glo® Substrate. Mix by inversion until the substrate is thoroughly dissolved.

**Product	Size	Cat. #**

Steady-Glo® Luciferase Assay System	10ml	E2510

Each system contains sufficient reagents for 100 assays

of 100µl each. Includes:

• 1 vial Steady-Glo® Luciferase Assay Buffer

• 10ml Steady-Glo Luciferase Assay Substrate (lyophilized)

• 1 Protocol
